# In vivo neurometabolic profiling in orthostatic tremor

**DOI:** 10.1097/MD.0000000000004848

**Published:** 2016-09-16

**Authors:** Julián Benito-León, Elan D. Louis, Virginia Mato-Abad, Ulrike Dydak, Juan Álvarez-Linera, Juan Antonio Hernández-Tamames, José Antonio Molina-Arjona, Norberto Malpica, Michele Matarazzo, Juan Pablo Romero, Álvaro Sánchez-Ferro

**Affiliations:** a Department of Neurology, University Hospital “12 de Octubre”; b Centro de Investigación Biomédica en Red sobre Enfermedades Neurodegenerativas (CIBERNED); c Department of Medicine, Complutense University, Madrid, Spain; d Department of Neurology, Yale School of Medicine; e Department of Chronic Disease Epidemiology, Yale School of Public Health; f Center for Neuroepidemiology and Clinical Neurological Research, Yale School of Medicine and Yale School of Public Health, New Haven, CT; g Neuroimaging Laboratory, Center for Biomedical Technology, Rey Juan Carlos University, Madrid, Spain; h School of Health Sciences, Purdue University, West Lafayette; i Department of Radiology and Imaging Sciences, Indiana University School of Medicine, Indianapolis, IN; j Department of Radiology, International Ruber Hospital; k Faculty of Biosanitary Sciences, Francisco de Vitoria University, Pozuelo de Alarcón, Madrid, Spain; l Research Laboratory of Electronics, Massachusetts Institute of Technology, Cambridge, MA; m HM CINAC, HM Hospitales, Madrid, Spain.

**Keywords:** case–control study, cerebellum, magnetic resonance imaging, orthostatic tremor, pathophysiology, proton magnetic resonance spectroscopy

## Abstract

The pathogenesis of orthostatic tremor (OT) remains unclear, although some evidence points to dysfunction in the brainstem or cerebellum. We used single voxel proton magnetic resonance spectroscopy (1H-MRS) (3 T) to investigate whether neurochemical changes underlie abnormal cerebellar or cortical function in OT. Fourteen OT patients and 14 healthy controls underwent 1H-MRS studies with voxels placed in midparietal gray matter and cerebellum (vermis and central white matter). Spectral analysis was analyzed using the software package LCModel (version 6.3). The absolute metabolite concentrations and ratios of total *N*-acetylaspartate + *N*-acetylaspartyl glutamate (NAA), choline-containing compounds, myoinositol, and glutamate + glutamine to creatine were calculated. In midparietal gray matter spectra, we found a significant decrease in the absolute concentration of NAA in OT patients versus healthy controls (7.76 ± 0.25 vs 8.11 ± 0.45, *P* = 0.017). A similar decrease in NAA was seen in the cerebellar vermis (7.33 ± 0.61 vs 8.55 ± 1.54, *P* = 0.014) and cerebellar white matter (8.54 ± 0.79 vs 9.95 ± 1.57, *P* = 0.010). No differences in the other metabolites or their ratios were observed. Reductions in both cerebral cortical and cerebellar NAA suggest that there is neuronal damage or loss in OT, raising the intriguing question as to whether OT is a neurodegenerative disease. Along with clinical history and electrophysio0logical examination, 1H-MRS could serve as a useful diagnostic aid for OT.

## 1 Introduction

Orthostatic tremor (OT), also known as “shaky legs syndrome”,^[1]^ is an enigmatic condition that was first reported in 1970 by Pazzaglia et al,^[2]^ although Heilman^[3]^ coined the term in 1984. The condition is characterized by tremor in the legs and the trunk, which begins upon standing. The tremor is accompanied by a sensation of unsteadiness, which is relieved with walking, while seated, or while lying down.^[4–6]^ OT may be a family of diseases, unified by the presence of regular, rapid lower limb tremor when standing, and further characterized by etiological and clinical heterogeneity.^[6,7]^ Gerschlager et al^[4]^ suggested the subdivision of OT into 2 broad groups—those with “primary OT” with or without postural arm tremor and those with “OT plus”, in whom there are additional associated movement disorders, mainly parkinsonism.

OT is not widely recognized by physicians who are not movement disorders experts, which often results in misdiagnosis, who then may be subjected to inappropriate or unnecessary tests and treatment. In fact, it is often misdiagnosed as essential tremor, Parkinson disease, restless leg syndrome, lumbar stenosis, and especially nonorganic (psychogenic) balance disorders.^[4,8,9]^ Of note, in the differential diagnosis, there are 3 other rare entities that one should keep in mind. One is orthostatic myoclonus, disorder that was first reported in 2007.^[10]^ This condition is characterized by unsteadiness during orthostatism and/or during gait and short-duration myoclonic bursts that occurred predominately with the assumption of an upright posture.^[10]^ Electromyogram (EMG) surface recording is essential for the diagnosis of this entity.^[10]^ Unlike OT, the bursts are shorter in duration, nonrhythmic, and irregular.^[10]^ The second one is low-frequency (4–6 Hz) leg tremor in some Parkinson disease, while they are standing.^[11,12]^ The response of this type of tremor to dopaminergic drugs is usually good.^[11,12]^ The third one is “pseudo-orthostatic tremor”, a levodopa-responsive entity, characterized by 6 to 7 Hz with sporadic subharmonics at 8 to 18 Hz, clinically manifested by standing tremor, preceding a parkinsonian syndrome.^[13]^

The pathogenesis of OT remains unclear, although clinical and neuroimaging data suggest a possible role of the brainstem or cerebellum.^[5,14–18]^ Furthermore, cerebellar outflow pathways are the target of high-frequency deep brain stimulators, which are an effective treatment in some OT patients.^[19,20]^ Of note is that in a recent case–control comparison, 16 OT patients scored less than 32 healthy matched controls in numerous cognitive tests, showing deficits in widespread cognitive domains including executive function, visuospatial ability, verbal memory, visual memory, and language.^[21]^ Hence, it is plausible that neurometabolic profiling could be altered in the cerebral cortex of OT patients.

Proton magnetic resonance spectroscopy (1H-MRS), a noninvasive technique, is widely used to assess the neurometabolic profile of neurological diseases, including tremulous disorders, such as Parkinson disease, dementia with Lewy bodies and essential tremor, or dystonia.^[22–25]^ The main metabolites detected by 1H-MRS are *N*-acetylaspartate + *N*-acetylaspartyl glutamate (NAA), creatine (Cr), choline-containing compounds (Cho), and myoinositol (ml).^[26]^*N*-acetylaspartate is mainly found within neurons, and a reduction in *N*-acetylaspartate reflects neuronal dysfunction or loss.^[26]^ Cr provides an energy buffer in the brain, and it includes Cr and phosphocreatine.^[26]^ As an internal reference, it is stable in different pathophysiological conditions.^[26]^ Cho is involved in both synthesis and breakdown of phospholipid membranes.^[26]^ The ml is a marker of glial cells and, therefore, its concentration is proportional with the extent of gliosis.^[26]^

To our knowledge, no published 1H-MRS data are available in OT patients. In order to provide new insights into the neurochemistry of OT, metabolite levels of NAA, Cr, ml, and glutamate + glutamine (Glx), as estimated by single voxel 1H-MRS, were assessed in 14 OT patients and compared to those of 14 age-matched and healthy controls. We hypothesized that there would be a reduction in NAA in the cerebellum in OT, and, possibly in the cerebral cortex.

## 2 Methods

### 2.1 Participants

Patients with OT were consecutively recruited from December 2011 to May 2013 from the outpatient neurology clinics of the University Hospital “12 de Octubre” in Madrid (Spain), a public hospital, which covers an area of more than 400,000 inhabitants. Four neurologists with expertise in movement disorders (JB-L, MM, JPR, and AS-F) examined these patients who were referred to the outpatient neurology clinics with a subjective feeling of unsteadiness when standing, which was absent while walking, seated, or supine. The neurological examination comprised a general neurological examination and the motor portion of the Unified Parkinson Disease Rating Scale (m-UPDRS).^[27]^ Mild parkinsonian signs were defined as present when any one of the following conditions was met: 2 or more m-UPDRS^[27]^ ratings = 1, or 1 m-UPDRS^[27]^ rating = 2, or the m-UPDRS rest tremor rating = 1. Diagnoses of OT were assigned by the 4 neurologists using the Consensus Statement on Tremor by the Movement Disorder Society^[28]^—that is, a suggestive clinical picture of OT (subjective feeling of unsteadiness during stance without problems when sitting and lying, and sparse clinical findings that are mostly limited to a visible and occasionally, only palpable fine amplitude rippling of the leg muscles when standing) that is confirmed by EMG recordings (i.e., synchronized leg tremor present only on standing).

A senior neuropsychologist (Verónica Puertas, see Acknowledgments), specializing in cognitive problems associated with movement disorders, performed a mental status examination on each patient and control applying the DSM-IV^[29]^ criteria and excluded those persons who had dementia. As described previously,^[21]^ each participant underwent a neuropsychological assessment, which included a detailed evaluation of cognitive function, with tests of attention and cognitive processing speed, executive function, visuospatial ability, verbal memory, visual memory, language, and mood.

Of 21 eligible OT patients, 7 were excluded from the final cohort. One 92-year-old woman with OT was excluded because she could not complete the magnetic resonance imaging (MRI) procedure due to her advanced age, another 81-year-old woman with OT suddenly died just before the MRI procedure, and 2 women (80 and 92 years) and 3 men (65, 66, and 77 years) with OT refused to undergo the MRI procedure. No healthy control was excluded due to incomplete neuropsychological evaluation or refusal to perform MRI. No participants were excluded because of major acute comorbidities, neurological comorbidities, dementia, or structural abnormalities on conventional MRI images.

OT cases were 1:1 frequency-matched with healthy controls. Frequency-matching was based on age and years of education.

Healthy controls were recruited either from relatives or friends of the health professionals working at the University Hospital “12 de Octubre” of Madrid (Spain) or among the relatives of patients who came to the neurological clinics for reasons other than OT (e.g., headache and dizziness). None reported having a first- or second-degree relative with OT or essential tremor. Each control underwent a standardized neurological examination performed by 1 of 2 neurologists (JPR and AS-F) to further rule out any neurological conditions, and by a neuropsychologist, as noted above.

As there is no validated tool to measure the severity of OT among patients, we assessed its impact on their health-related quality of life (HRQoL) by means of the Spanish version of EuroQol-5 dimension (EQ-5D), a standardized instrument developed by the EuroQol group, a consortium of investigators in Europe.^[30]^ The EQ-5D was chosen as the generic HRQoL measure given its widespread use and its apparent applicability to other tremor disorders such as Parkinson disease^[31]^ and essential tremor.^[32]^ EQ-5D consists of 2 parts: the EQ-5D descriptive system and the EQ visual analog scale (EQ VAS).^[30]^ The EQ-5D descriptive system comprises 5 dimensions: mobility, self-care, usual activities, pain/discomfort, and anxiety/depression.^[30]^ Each dimension has 3 levels (no problems, some problems, and major problems) and together results in a numeric value that defines a health state. EQ-5D scores range between −0.594 and 1 (full health).^[30]^ The EQ VAS records the respondent's self-rated health on a 20-cm vertical, visual analog scale with endpoints labeled “the best health you can imagine” (100) and “the worst health you can imagine” (0).^[30]^

According to a recently published comorbidity score developed in ambulatory care settings, a comorbidity index was calculated.^[33]^ The presence of several conditions (atrial fibrillation, nonmetastatic cancer, metastatic cancer, chronic obstructive pulmonary disease, depression, dementia, diabetes, epilepsy [treated], heart failure, myocardial infarction, psychiatric disorders, renal disease, and stroke) resulted in the assignment of more points than others, and the score ranged from 0 to 28 (i.e., all conditions present).^[33]^

### 2.2 1H-MRS acquisition

MRI was performed on all OT patients and healthy controls on a 3T system (GE Signa 3T HDxt scanner, General Electric Medical Systems, Milwaukee, WI) using an 8-channel phased array coil. The MRI protocol included a 3-dimensional T1-weighted Spoiled Gradient Recalled with a Repetition Time = 9.24, Echo Time = 4.132 ms, Inversion Time = 500 ms, Number of Excitations = 1, acquisition matrix = 256 × 256, full brain coverage, resolution = 0.9375 × 0.9375 × 1 mm^3^, and flip angle = 12. The 1H-MRS protocol consisted of 3 Point Resolved Spectroscopy (PRESS) acquisitions with TR = 2000 ms and TE = 35 ms. The following specific voxels were selected:

1. Cerebellar gray matter. The voxel (nominal size = 1.5 × 1.5 × 1.8 cm^3^) was placed in the vermis. Aside from a small proportion of white matter (around 20% of the total volume) and cerebrospinal fluid (CSF) (around 10%), the voxel contained mainly gray matter (around 70%). Due to having to adjust the voxel size minimally in some subjects to match their head size, the effective voxel size (mean ± standard deviation [SD]) was 4 ± 0.28 cm^3^.
2. Cerebellar white matter (nominal voxel size: 1.5 × 1.5 × 1.3 cm^3^). Besides a small proportion of gray matter (around 10%), the voxel contained nearly exclusively white matter (around 90%). The mean voxel size was 3 ± 0.25 cm^3^.
3. Midparietal gray matter (nominal voxel size = 2.2 × 2.2 × 2.2 cm^3^). The mean voxel size was 10 ± 0.19 cm^3^ and mainly contained gray matter (around 65%), with some white matter (around 20%) and CSF (around 15%). Our motivation for including this particular voxel was the recent finding of widespread cognitive impairment in OT.^[21]^ In addition, the same voxel location has been used to quantify metabolites in other diseases that present with cognitive impairment.^[34,35]^

**Figure 1:**
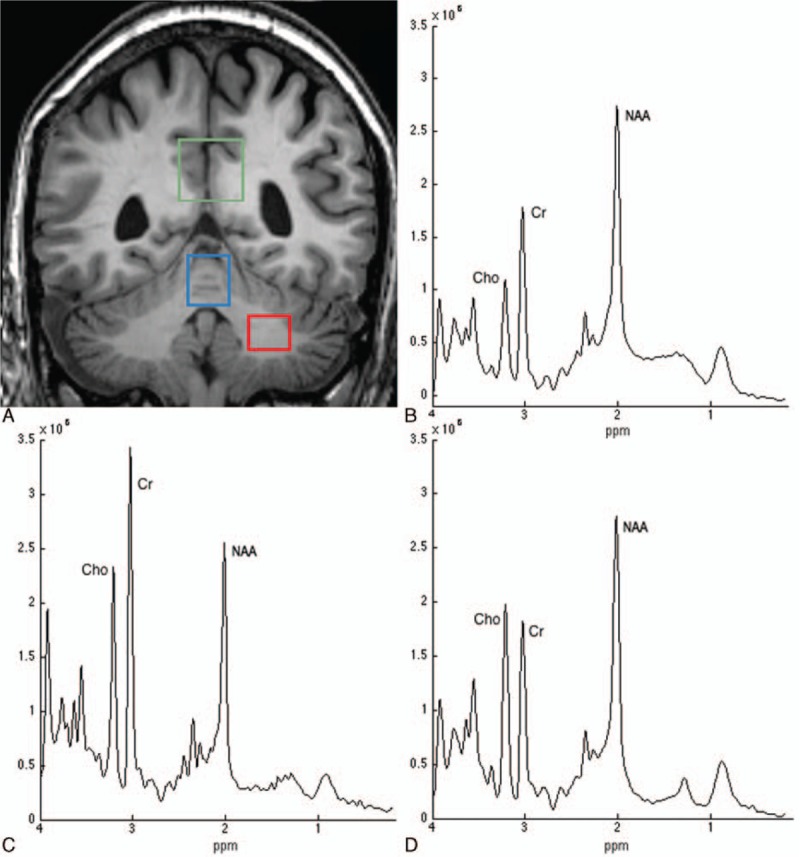
T1-weighted magnetic resonance image showing the region-of-interest locations used for proton magnetic resonance spectroscopy of midparietal gray matter (green voxel), cerebellar vermis (blue voxel), and the central cerebellar white matter (red voxel) (A). The spectra shown were obtained from a voxel in the midparietal gray matter (B), cerebellar vermis (C), and the central cerebellar white matter (D) of a 62-year-old orthostatic tremor patient. The labeled resonances are those of *N*-acetylaspartate + *N*-acetylaspartyl glutamate (NAA), total creatine (Cr), and total choline-containing compounds (Cho). The spectra are plotted on the same vertical intensity scale normalized to background noise. ppm = parts per million. The absolute concentration of NAA is reduced in this patient compared to the control group in the 3 voxels, whereas there is minor change in either Cho or Cr.

Voxel placements and representative spectra from each location are depicted in Fig. 1. All MRI/1H-MRS acquisitions and image postprocessing (see below) were performed by a neuroradiologist (JA-L) and a physicist (VM-A) who were blinded to the clinical diagnoses.

### 2.3 1H-MRS postprocessing

All spectra were analyzed using the sofware package LCModel (version 6.3) (Stephen Provencher Inc., Oakville, ON, Canada).^[36]^ The LCModel method automatically performs phase adjustments, frequency alignment, baseline subtraction, and eddy current correction. Relative metabolite concentrations (and their uncertainties) are estimated by fitting the spectrum to a linear combination of “basis spectra” of each individual metabolite, obtained from solutions acting as concentration references for the in vivo acquisitions. For this study, a basis set provided by the LCModel software for a 3-T PRESS acquisition with TE = 35 ms was used. The unsuppressed water spectrum is then used to normalize the initial fit to generate a first estimate of metabolite concentration in the tissue. LCModel defines the concentrations of the pertinent metabolites by scaling the relative areas and chemical shifts across the 2 sets of spectra. The fitting of the spectral peaks was thus achieved with a priori knowledge of their actual characteristics.

The main brain metabolites of interest were the NAA peak at 2.0 ppm, the Cr peak at 3.0 ppm, the Cho peak at 3.2 ppm, the ml peak at 3.5 ppm, and the Glx peak at 2.2 to 2.4 ppm.

To define a criterion for rejection of poor-quality spectra, the Cramer Rao lower bounds (given as SD% value by the LCModel) were used. Only those spectra with a SD% < 20 were included in the study. In all these spectra, the linewidth, obtained during the shimming process, was (on average) 5.75, 6.6, and 6.5 Hz for gray matter of the cerebellum, white matter of the cerebellum, and midparietal gray matter, respectively, with no significant mean differences between the groups.

Finally, metabolite concentrations obtained from LCModel were corrected for gray and white matter and CSF content.^[37]^ The 3-dimensional high-resolution T1-weighted images were segmented into gray matter, white matter, and CSF using SPM-8 toolbox (www.fil.ion.ucl.ac.uk/spm/) to determine the tissue composition of each voxel of interest. Metabolite quantification was adjusted for partial volume effects as previously described.^[37]^

All procedures were approved by the ethical standards committees on human experimentation at the University Hospital 12 de Octubre (Madrid). Written (signed) informed consent was obtained from all enrollees.

### 2.4 Statistical analyses

All statistical analyses were performed using SPSS, version 21.0 (IBM Corp., North Castle, NY). Mean scores (age and neuropsychological variables) were compared using 2 independent sample *t* tests for continuous and normally distributed data, and Mann–Whitney *U* test for nonnormally distributed data, where appropriate. The χ^2^ test was used to analyze differences in sex distribution. The peaks of NAA, Cho, ml, and Glx, as well as NAA/Cr, Cho/Cr, and Glx/Cr, were calculated for each subject and location and compared using 2 independent sample *t* tests because they were all normally distributed. Correlations between age, years of education, age at onset, disease duration, 17-item Hamilton Depression Rating Scale total score, comorbidities, EQ-5D score, cognitive domains, and 1H-MRS metabolites were determined using Pearson correlation coefficients.

## 3 Results

Clinical details of the OT patients are provided in Table 1. All 14 OT patients were right-handed (mean age 65.0 years, range 37–81 years). There was a female preponderance (N = 12, 85.7%) with a mean age of onset of 55.6 years. All patients presented with unsteadiness that occurred within seconds of standing and was relieved by walking or sitting. The symptoms of unsteadiness coincided with the appearance of tremor in the legs and trunk. Nine (64.3%) patients presented with primary OT and 5 (35.7%) had additional neurological features (mild parkinsonian signs). Ten (71.4%) patients reported a progressive course. Structural brain MRI was unremarkable in all 14 patients, none had cerebellar atrophy. Routine blood and chemistry tests including thyroid function tests, serum protein electrophoresis, and vitamin B12 levels were also in the normal range in all 14 patients. Before MRI procedure, all 14 OT patients underwent electromyographic analysis of their leg tremors. This revealed a synchronous 10- to 18-Hz leg tremor that was present only on standing. No patients were being treated with medication for OT (i.e., clonazepam, dopaminergic drugs, gabapentin, or barbiturates).

**Table 1:**
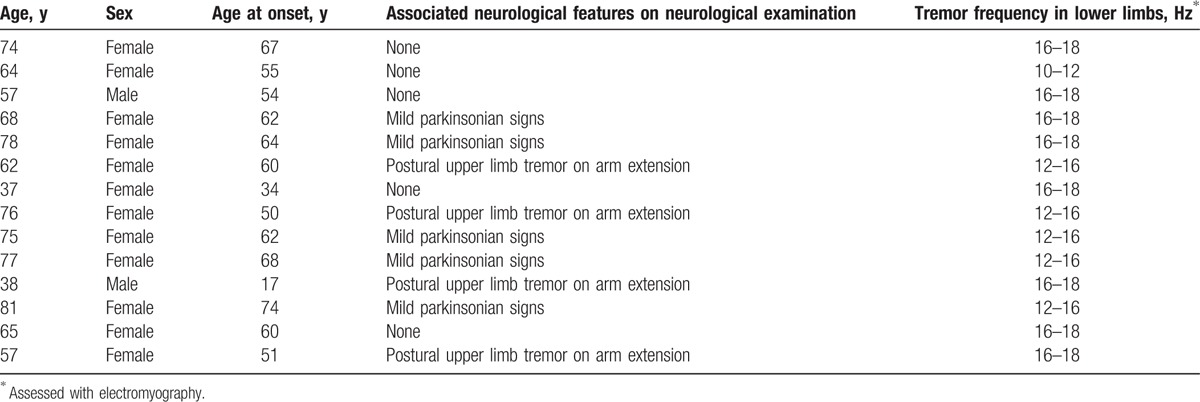
Demographic and clinical characteristics of 14 OT patients.

The 14 right-handed OT patients (12 women and 2 men) were compared with 14 right-handed healthy controls (9 women and 5 men). The OT patients did not differ to a significant degree from the healthy controls in terms of age, sex, years of education, comorbidity index, and depressive symptoms (Table 2).

**Table 2:**
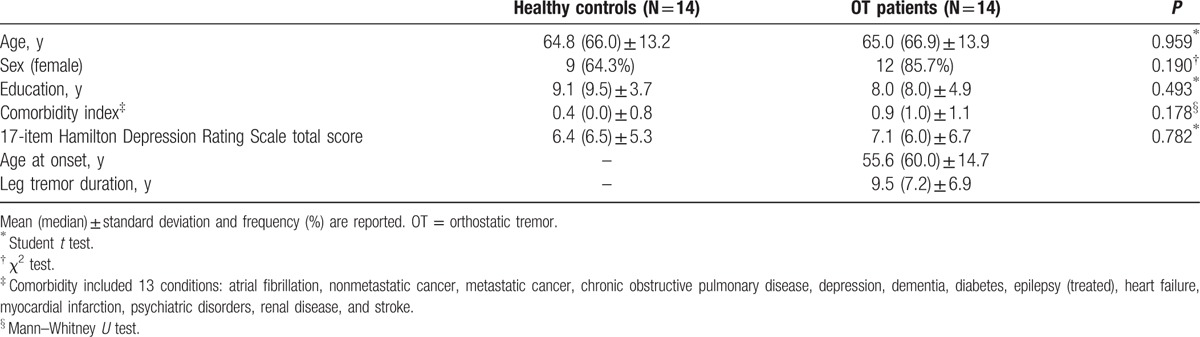
Comparison of demographic and clinical variables of OT patients versus healthy controls.

### 3.1 1H-MRS results

All metabolite spectra included in this study had a SD% ≤ 8% for NAA, ≤9% for Cho and Cr, and ≤14% for ml. The mean NAA SD% in midparietal gray matter was 2.21% (SD = 0.43) and 2.13% (SD = 0.35) in OT patients and healthy controls, respectively (Mann–Whitney *U* test, *P* = 0.715). In the cerebellar vermis voxel, the mean NAA SD% was 3.54% (SD = 0.66) and 3.47% (SD = 0.64) in OT patients and healthy controls, respectively (Mann–Whitney *U* test, *P* = 0.786). Finally, in the central cerebellar white matter voxel, the mean NAA SD% was 5.36% (SD = 0.63) and 5.07% (SD = 1.38) in OT patients and healthy controls, respectively (Mann–Whitney *U* test, *P* = 0.210).

The means and SDs of the metabolite ratios are presented in Table 3. In midparietal gray matter spectra, we found a significant decrease in the absolute concentration of NAA in OT patients versus healthy controls (7.76 ± 0.25 vs 8.11 ± 0.45, *P* = 0.017). A similar difference was also seen in both the cerebellar gray (vermis) (7.33 ± 0.61 vs 8.55 ± 1.54, *P* = 0.014) and white matter (8.54 ± 0.79 vs 9.95 ± 1.57, *P* = 0.010). No significant differences in the other metabolites or their ratios were observed (Table 4).

**Table 3:**
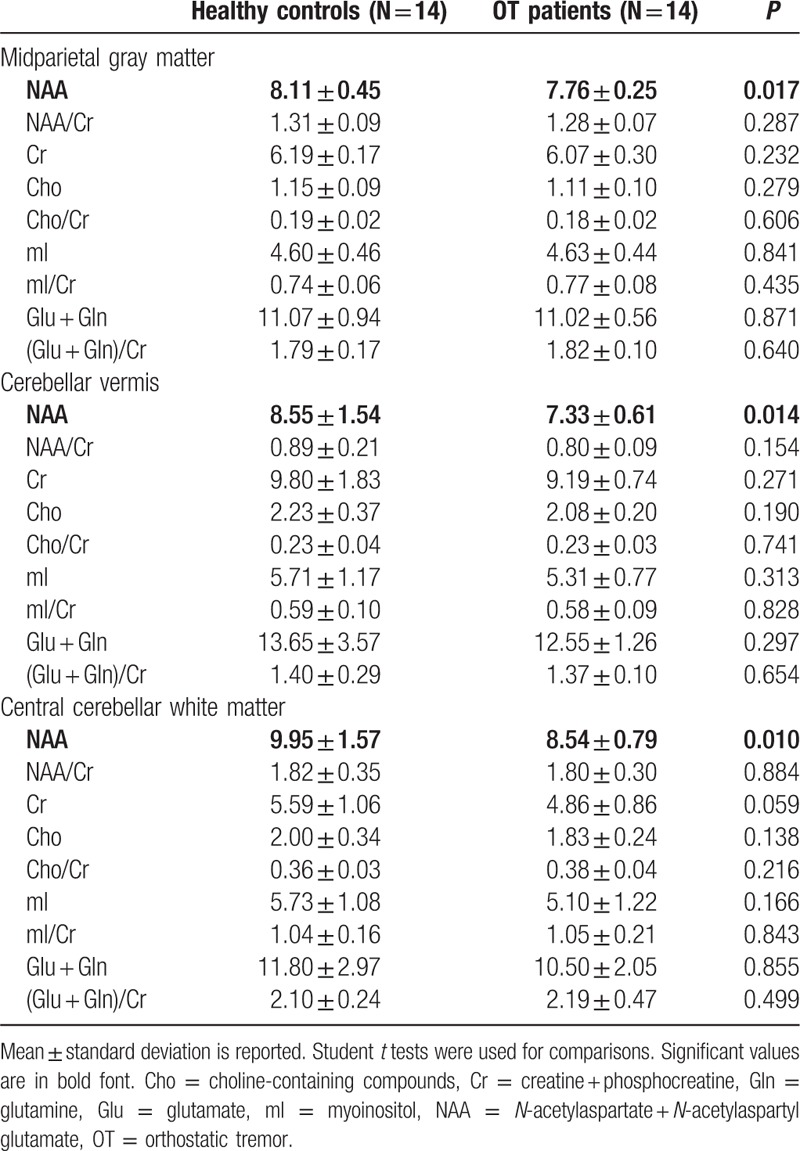
Absolute concentrations of metabolites and metabolic ratios in OT patients versus healthy controls.

**Table 4:**
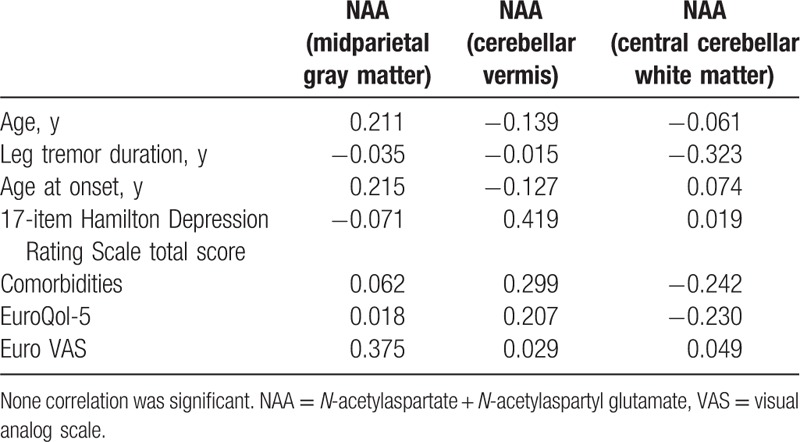
Pearson moment correlations between the absolute NAA concentration and demographic and clinical variables in 14 OT patients.

### 3.2 Relationships between NAA, demographic and clinical variables

These correlations were calculated in OT patients only, and only for the metabolite (NAA), which was different in OT patients versus control subjects (Table 3). We did not find any significant correlation of NAA with demographic and clinical variables (Table 4).

## 4 Discussion

We demonstrated a reduction in cerebral cortical and cerebellar NAA concentration in OT patients, suggesting the presence of neuronal damage or loss in these regions.

Very little is known about the pathogenesis of OT; however, it is not typically included among the neurodegenerative disorders. There is some evidence of a potential role of the nigrostriatal dopaminergic system in the pathogenesis of OT. For example, using 123I-FP-CIT single-photon emission computed tomography, the dopaminergic system was affected in a group of 11 OT patients, although to a lesser extent than in Parkinson disease.^[38]^ When compared to a group of 12 Parkinson disease patients, tracer uptake in OT patients was significantly higher and more symmetrical, and the caudates and putamens were equally affected.^[38]^ However, these findings are not universal, and other functional imaging studies have showed intact dopaminergic systems.^[39,40]^

We demonstrated a reduction in cerebral cortical and cerebellar cortical viability. While it is possible that the observed metabolic abnormality in the cerebral cortex and cerebellum is not a primary pathological event in OT, these data raise the possibility that OT could be a neurodegenerative disease. Other clinical data provide some additional support for this possibility. First, OT is a progressive disorder with increasing disability, suggesting that the underlying pathological process may not be static.^[41,42]^ Also, patients who initially present with isolated OT often later develop additional neurological signs, including parkinsonism.^[42]^

Reduction in cerebral cortical and cerebellar NAA concentration could be explained by neuronal damage or loss, but it could alternatively be interpreted in terms of changes in energy demand.^[43,44]^ A gradual reduction in NAA in the visual cortex has been reported, using 3-^[43]^ or 7-T^[44]^ 1H-MRS, during visual stimulation, with a progressive recovery after the stimulation ended. Baslow et al^[43]^ proposed that the reduction of NAA following neuronal activation was related to activation-induced neuronal release of NAA in the extracellular compartment to control water distribution. An alternative explanation was put forward by Mangia et al,^[44]^ who proposed that NAA change was linked to the involvement of this metabolite in the Krebs cycle and in oxidative metabolism. Thus, the decrease in NAA in OT might be linked to a higher energy demand of neurons due to the increased EMG activity resulting from tremor. In line with this hypothesis, NAA would be decreased in the areas that represent the nodes of the network involved in producing the tremor (e.g., the cerebellum).

The study should be interpreted within the context of several limitations. First, the sample size was relatively small. Given the low incidence and prevalence of the disease, the OT literature generally comprises studies with small sample sizes. Although there are no available epidemiological data, in the follow-up evaluation of the Neurological Disorders of Central Spain study,^[45]^ we detected only 1 patient with OT in a cohort of approximately 4000 elderly subjects (data not published). Despite this limitation, our study was able to detect significant case–control differences. Second, the patients in the present study may represent a selected group of OT patients (i.e., patients seen in selected outpatient clinics). However, in Spain, health care is fully state-subsidized, and community-dwelling OT patients are mainly seen by hospital-based and hospital-associated neurologists. Third, the recruited sample was quite heterogeneous, including primary and OT plus cases. However, our aim was to examine whether OT patients in general had altered spectroscopic data when compared with matched healthy controls.

Despite these limitations, this research represents the first application of 1H-MRS to the study of OT and the first to demonstrate a metabolic abnormality in the brains of OT patients. This and other future 1H-MRS studies might provide a novel method to understand the pathophysiological mechanism of OT. Further, our findings suggest that 1H-MRS could prove useful as a useful diagnostic tool in OT, along with the clinical history and electrophysiological examination.

## Acknowledgments

We acknowledge Dr Verónica Puertas for her assistance with the project.
